# Is there still a role for thyroid scintigraphy in the workup of a thyroid nodule in the era of fine needle aspiration cytology and molecular testing?

**DOI:** 10.12688/f1000research.7880.1

**Published:** 2016-04-27

**Authors:** Rodrigo Moreno-Reyes, Aglaia Kyrilli, Maria Lytrivi, Carole Bourmorck, Rayan Chami, Bernard Corvilain

**Affiliations:** 1 Department of Nuclear Medicine, Erasme University Hospital, Université Libre de Bruxelles, Brussels, Belgium; 2 Department of Endocrinology, Erasme University Hospital, Université Libre de Bruxelles, Brussels, Belgium

**Keywords:** Thyroid, scintigraphy, AFTN

## Abstract

Thyroid scintigraphy is now rarely used in the work-up of a thyroid nodule except in the presence of a low TSH value. Therefore, autonomously functioning thyroid nodules (AFTNs) with a normal TSH value are diagnosed only in the rare medical centers that continue to use thyroid scan systematically in the presence of a thyroid nodule. In this review, we discuss the prevalence of AFTN with a normal TSH level and the possible consequences of performing fine needle aspiration cytology (FNAC) in an undiagnosed AFTN. We also discuss the risk of malignant AFTN which may be higher than previously stated.

## Introduction

Thyroid nodules are a very common problem in adults. Their prevalence increases with age and may reach 50% by the age of 65 years ^1– 2^. Thyroid scintigraphy is the only technique that permits evaluation of the functional characteristics of a nodule. Two radionuclides are mainly used for the evaluation of patients with thyroid nodules: ^99m^TcO4 ^-^ and ^123^I. ^123^I is both concentrated and organified within the gland, whereas ^99m^TcO4 ^-^ is only concentrated. According to the 2015 American Thyroid Association (ATA) guidelines, if a thyroid scan is performed, ^123^I should be preferred over ^99m^TcO4 ^-^^3^, but this preference is not justified for the European Association for Nuclear Medicine ^4^. Thyroid nodules are classified according to their ability to take up the isotope compared to that of the extranodular tissue. A cold nodule (hypofunctional) has reduced tracer uptake, a warm nodule (isofunctional) has tracer uptake roughly equivalent to the non-nodular tissue, and a hot nodule (hyperfunctional) has increased tracer uptake. The term autonomously functioning thyroid nodules (AFTNs) is frequently used as synonymous for hot nodules because they are characterized by their capacity to grow and produce thyroid hormones in the absence of thyroid-stimulating hormone (TSH) ^5^. In the presence of a hot nodule and a normal level of TSH, the autonomous function can be formally demonstrated by administration of a suppressive dose of thyroid hormone and showing that it does not affect the function of the nodule (persistence of increased radionuclide uptake). This test is rarely used in clinical practice. The vast majority of nodules are hypofunctioning (cold nodules), whilst a minority is hyperfunctioning. The thyroid scan should always be compared to thyroid ultrasound images to be sure that the abnormality observed on the scan corresponds to a thyroid lesion. AFTNs account for 5–10% of palpable nodules and up to 20% in regions with iodine deficiency ^6– 9^. Gain-of-function mutations of the TSH receptor (TSHR) or of the α subunit of the stimulating G-protein (Gsα) are the main causes of AFTNs. The frequencies of Gsα and TSHR mutation in AFTNs reach 5% and 60%, respectively. The mutations responsible for the remaining cases remain unknown but are also probably involved in constitutive activity of cAMP cascade (e.g. other G-protein subunits, adenylyl cyclase, phosphodiesterase, and protein kinase A). Other pathways may also play a role in the pathogenesis of AFTNs like vascular growth factors ^10^, AMPK signaling pathway ^11^, and miRNA cascades ^12^. Thus far, more than 40 TSHR mutations have been reported ^13– 15^. It is generally accepted that the risk of cancer is around 5% in a cold nodule and extremely low in AFTNs, which therefore do not require further investigation ^16^. Driven by studies from areas with normal iodine intake, mainly North America, thyroid scintigraphy is now rarely used in the management of thyroid nodules in the presence of a normal TSH value. This strategy is based on the assumption that AFTNs are uncommon and that TSH levels are always subnormal in the presence of an AFTN and therefore that a normal TSH value rules out the presence of an AFTN. Consequently, the ATA and the European Thyroid Association (ETA) guidelines recommend considering radionuclide scanning in patients with thyroid nodules only if their TSH is low ^3, 17^. For the ETA, a thyroid scan may also be considered in the presence of a multinodular goiter in areas with insufficient iodine supply. Despite case reports of hot nodules that have turned out to be malignant, most groups agree that AFTNs do not necessitate fine needle aspiration cytology (FNAC) based on their very low risk of cancer. An additional reason to avoid FNAC of AFTNs is the risk of obtaining equivocal results (follicular lesion of unknown significance or FLUS), which tends to prompt surgery ^18^. However, the risk of malignancy in an AFTN has not been clearly quantified in the literature. Independent of a better estimation of the risk of cancer, diagnosis of AFTN by a thyroid scan will change the follow up that will mainly consist of prevention, detection, and adequate treatment of thyroid dysfunction. The rate of development of thyrotoxicosis in patients with hyperfunctioning adenomas who are euthyroid initially is about 4% per year ^19– 21^. It is important to point out that in patients with an AFTN, even a small increase in iodide supply leads to increased thyroid hormone synthesis and will accelerate the development of thyrotoxicosis ^22, 23^.

In this review, we discuss whether there remains a place for thyroid scintigraphy in the workup of a thyroid nodule in the era of FNAC and molecular testing. To answer this question, we address the following points:

## 1. Does normal TSH exclude a hyperfunctioning thyroid nodule?

**Figure 1.**
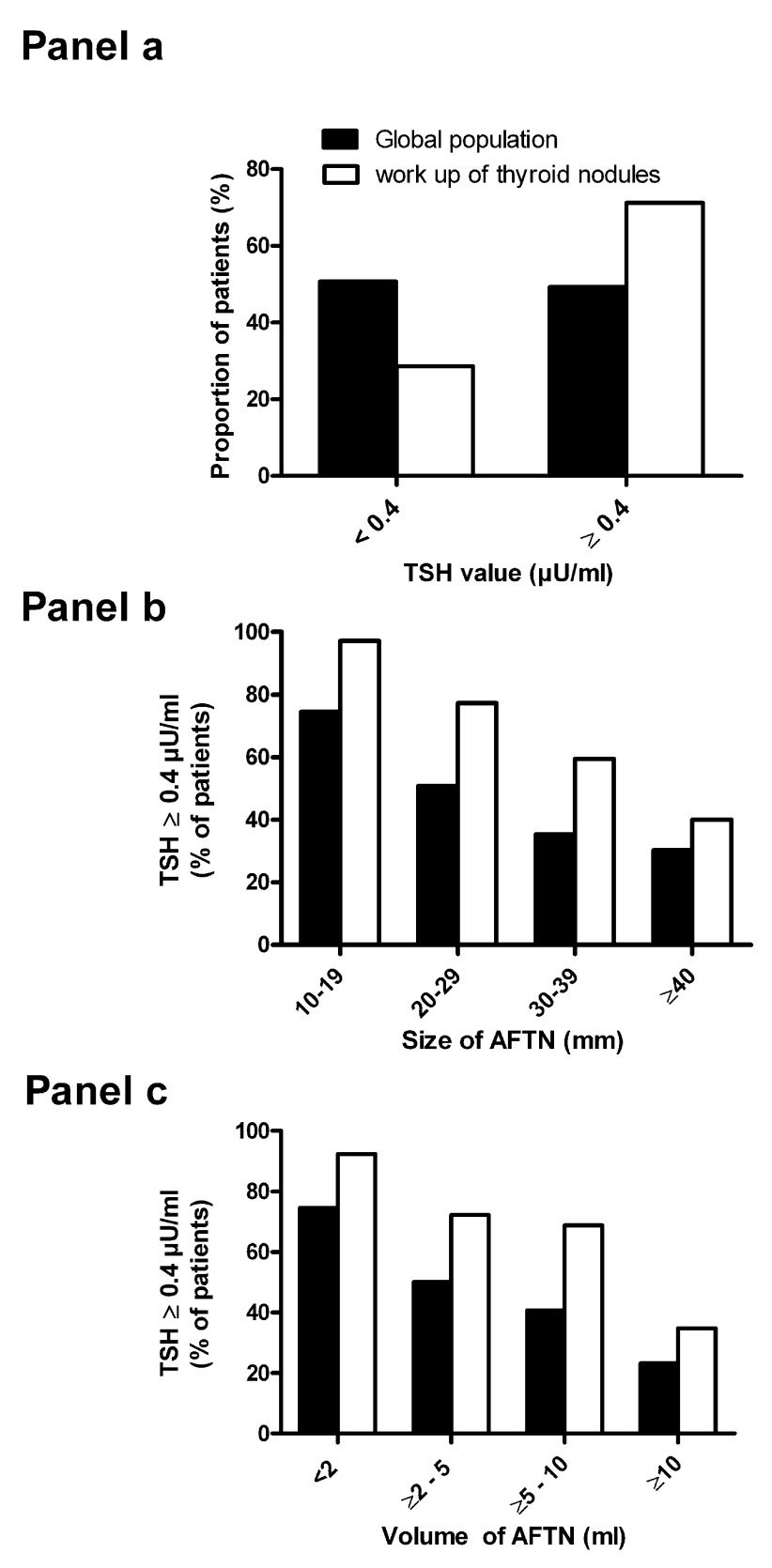
TSH level in patients with an autonomously functioning thyroid nodule. **Panel a**: Proportion of patients with an autonomously functioning thyroid nodule (AFTN) and a normal thyroid-stimulating hormone (TSH) level (Black bars: global population; white bars: subpopulation in which the AFTN was discovered in the workup of a thyroid nodule). **Panels b** and **c**: Proportion of patients with a normal TSH level according to the size of the nodule; main diameter ( **panel b**) or volume ( **panel c**). (Black bars: global population; white bars: subpopulation in which the AFTN was discovered in the workup of a thyroid nodule).

**Figure 2.**
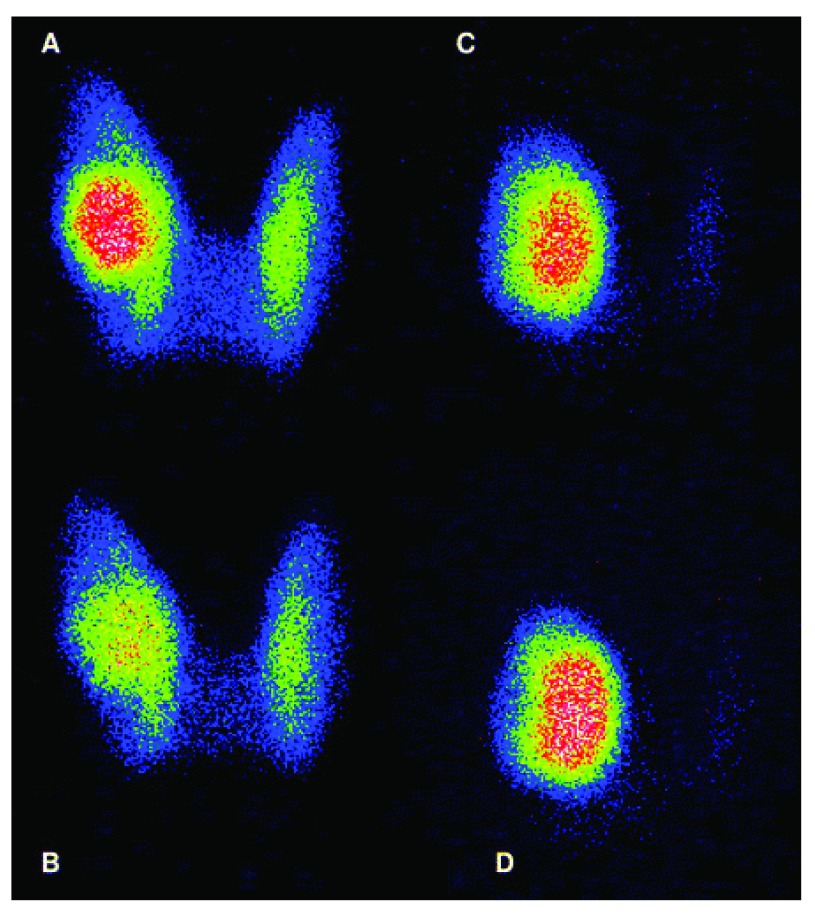
Iodide perchlorate discharge test in two patients with an autonomously functioning thyroid nodule Iodide perchlorate test was used to evaluate organification defect in patients with an autonomously functioning thyroid nodule (AFTN). A time 0, 26MBq ^123^I was administered orally with 500 µg stable iodide. Three hours later, a first scintigraphy was acquired, followed immediately by the oral administration of perchlorate. One hour later, a second scintigraphy was obtained. After correction for radioisotope decay, discharge can be evaluated by comparing the counts before and after perchlorate. Thyroid scan in a patient with a positive discharge test before ( **A**) and after ( **B**) perchlorate. Thyroid scan in a patient with a negative perchlorate test before ( **C**) and after ( **D**) perchlorate.

The ATA and the ETA guidelines recommend considering radionuclide scanning in patients with thyroid nodules only if the TSH level is low ^3, 17^. However, this assertion comes from small clinical studies ^24^ or expert opinions ^25^. In the absence of thyroid scintigraphy in the workup of a patient with a thyroid nodule and a normal TSH level, there is a risk of performing FNAC in an unsuspected AFTN. Until recently, the proportion of patients with an AFTN and a normal TSH level was unknown. In 2014, we published a study of 368 patients, which demonstrated that more than 70% of patients with an AFTN referred to our hospital for the workup of a thyroid nodule had a normal TSH level ^26^. The proportion of patients with subclinical hyperthyroidism increased with the size of the nodule and reached 50% or more only for patients with a nodule size above 3 cm ( Figure 1). A recent meta-analysis confirmed that TSH is not an effective tool to detect or exclude an AFTN ^27^. However, the majority of these studies came from European countries with a history of past or present iodine deficiency ^28– 30^. Therefore, we cannot claim that this observation is also valid for patients from areas with sufficient iodine intake. Another limitation of these studies is the fact that thyroid scans were generally performed using ^99m^TcO4 ^-^ rather than ^123^I and that some nodules may appear “hot” on ^99m^TcO4 ^-^ but are actually cold on ^123^I. This discordance is observed in 5-10% of nodules and up to 30% of them may be malignant ^31^. The absence of hyperthyroidism in a large proportion of patients with an AFTN is not surprising, as the majority of the described TSHR mutations only activate the cyclic AMP cascade that is involved in the stimulation of iodide transport but not in its organification ^13– 15, 32^. Iodide organification and thyroid hormone synthesis are stimulated by the Ca ^2+^−IP3 cascade ^32^. This cascade is constitutively activated only by a minority of TSHR mutations. This explains why an organification defect is observed in the majority of AFTN cases ^33^. In this latter study, the iodide perchlorate discharge test was used to diagnose impaired iodide organification. Perchlorate is a competitive inhibitor of iodide trapping. In cases of normal organification, perchlorate does not cause significant changes in the iodide content of the thyroid, since most iodide is organified and cannot be released. In cases of organification defect, administration of perchlorate causes a decrease in thyroid iodide content. Examples of AFTN with or without iodide organification defect are shown in Figure 2.

In our study, 60% of patients with an AFTN had an organification defect as revealed by the perchlorate discharge test ^33^. Some differences in thyroid hormone level were observed between the two groups, but our population was too small to make a definitive conclusion. No genetic data were obtained, but our hypothesis was that AFTNs harboring a TSHR mutation that activates only the cAMP cascade had an organification defect, while AFTNs harboring a TSHR mutation that activates both the cAMP and the Ca ^2+^−IP3 cascade had a more severe phenotype owing to more efficient thyroid hormone synthesis. Not only is the diversity in clinical behavior of AFTN probably caused by differences in causal TSHR mutations but it may also reflect the differences in cellular density from one nodule to another owing to various decreases in colloid space and the difficulty of estimating the actual weight of the active nodule ^13^. Whatever the reason for the persistence of a normal TSH level, on the basis of a “TSH only” screening, as recommended by the vast majority of international guidelines, more than 50% of patients with AFTN may undergo unjustified FNAC in the workup of a thyroid nodule.

## 2. What is the ultrasound appearance of an AFTN?

If thyroid scan is not used in the workup of a thyroid nodule, ultrasound malignancy criteria are now current practice to establish the necessity of FNAC ^34^. These criteria include the presence of microcalcifications, nodule hypoechogenicity, irregular margins, intranodular vascularity, and a nodule with a shape taller than it is wide. As thyroid scintigraphy is no longer systematically performed in the workup of a thyroid nodule, the literature is inconclusive on the ultrasound appearance of an AFTN. One uncontrolled retrospective study reported that more than 30% of AFTNs may have a suspicious feature on ultrasound (mainly a hypoechoic aspect and intranodular vascularization) ^35^. Another study reported the presence of microcalcifications in more than 50% of AFTN cases ^36^. Perinodular and intranodular signals evaluated by color flow Doppler sonography are significantly higher in AFTNs than in cold nodules ^37, 38^. These findings in the absence of a thyroid scan always lead to the performance of FNAC. The exact percentage of patients with an unsuspected AFTN who deserve FNAC according to the ultrasonographic appearance of the nodule needs to be evaluated in a prospective study.

## 3. What is the risk of obtaining a cytological report of an indeterminate follicular lesion that may lead to unnecessary surgery when a FNAC is performed on an unsuspected AFTN?

Once again, as thyroid scintigraphy is no longer systematically performed and as an AFTN does not warrant FNAC, data in the literature on the cytological aspect of an AFTN are rare. When FNAC is performed on a known AFTN, it is generally accepted that the risk of obtaining a cytological report of a FLUS is not negligible, but until now only small uncontrolled studies have been published ^18, 36, 39^. No data exist for assessing the risk of malignancy in an AFTN when an FNAC is read as an indeterminate follicular lesion. The fact that a thyroid scan is no longer used in the workup of a thyroid nodule in euthyroid patients increases the likelihood of performing FNAC on an unsuspected AFTN and the risk of performing thyroid surgery for benign lesions, although removal of a hot nodule may eliminate a long-term risk of developing hyperthyroidism. A prospective study of the results of FNAC when performed in an AFTN is needed to quantify more precisely the risk of surgery in such patients.

## 4. Can molecular testing improve the diagnosis of thyroid FNA specimens from AFTN?

Molecular testing for miRNA, mRNA, and DNA on FNA specimens improves the diagnosis of thyroid nodules with indeterminate cytology ^40– 44^. The most advanced multigene molecular panels provide both high positive predictive value and high negative predictive value for cancer detection in thyroid nodules, and in the near future they will probably eliminate indeterminate cytologic diagnosis of thyroid nodules. These techniques have never been studied for suspicious cytology obtained in an AFTN. If they prove to be reliable in an AFTN, molecular testing will also decrease the risk of unnecessary surgery in cases of indeterminate cytology in an AFTN. The cost effectiveness of performing a thyroid scan to avoid an unnecessary FNAC in an AFTN or to skip the thyroid scan and perform molecular testing in case of FNAC with indeterminate cytology in an AFTN is not known. This will depend on the cost of molecular testing (which will probably decrease in the next few years), the prevalence of AFTN (higher in areas where dietary iodine is or was insufficient), and the as yet unknown risks of obtaining indeterminate cytology in an AFTN.

## 5. What is the risk of cancer in an AFTN?

It is generally accepted that the risk of cancer in an AFTN is very low. However, the risk may be higher than generally believed, and in a recent review based on retrospective surgical series, the risk was estimated at 3%, i.e. nearly the same as for a cold nodule (5%) ^38^. The risk of thyroid microcarcinoma may reach 30% in autopsy studies ^45^. Therefore, there is a risk of overestimation of thyroid cancer in patients with AFTN owing to the high prevalence of incidentally found thyroid microcarcinoma at surgery. The possibility that high incidences of cancer in some series may result from misdiagnosis of malignancy was also suggested ^46^. There are no studies specifically designed to assess the risk of malignity of AFTNs as a primary outcome. There are many published case reports but with the obvious bias that only rare cases are reported. In some studies, tumor size is not reported and it is not clear if the observed cancer is an incidental microcarcinoma outside or within the AFTN, or within another nodule in the case of a multinodular goiter ^47– 55^. The risk of thyroid cancer within an AFTN seems independent of the level of TSH. The huge variation of the risk of malignancy within AFTNs between the different studies reported in Mirfakhraee *et al*.’s review (between 0% and 12.5%) raises the question of potential bias and underlines the need for prospective studies ^38^.

## Conclusion

Thyroid scan is now used in the workup of thyroid nodules only in the presence of a low serum TSH. This strategy is not appropriate, at least in areas with a present or past iodine deficiency. In these areas, several studies have demonstrated that up to 70% of patients with an AFTN may have a normal TSH value. This does not mean that thyroid scan must be performed in every patient with a thyroid nodule but only in those with an indication of FNAC, i.e. nodule above 10–15 mm of diameter. We believe that before giving up the use of thyroid scan, prospective studies must be done on the reliability of FNAC and molecular testing in an AFTN and on the risk of thyroid cancer in an AFTN.
